# Licorice in nephropathy treatment: phytochemical compositions and pharmacological mechanisms

**DOI:** 10.3389/fphar.2025.1672643

**Published:** 2025-11-21

**Authors:** Ya-Long Feng, Le Shui, Yin-Xuan Jiang, Yan-Ni Wang, Lin Chen, Hua Chen, Wen-Bo Wang

**Affiliations:** 1 Department of Life Science, Xianyang Normal University, Xianyang, Shaanxi, China; 2 Department of Clinical Research, Xianyang Hospital of Yan’an University, Xianyang, Shaanxi, China; 3 Department of Pharmaceutical Engineering, College of Biology Pharmacy and Food Engineering, Shangluo University, Shangluo, Shaanxi, China; 4 College of Pharmacy, Ningxia Medical University, Yinchuan, Ningxia, China

**Keywords:** licorice, nephropathy, chronic kidney diseases, *Glycyrrhiza uralenssis* fish., *Glycyrrhiza inflate* bat., *Glycyrrhiza glabra* L

## Abstract

Nephropathy refers to a wide range of kidney dysfunction and is a highly prevalent condition worldwide, often associated with several disorders, including inflammation, oxidative stress, apoptosis, epithelial-mesenchymal transition (EMT), autophagy, and the deposition of extracellular matrix (ECM). Licorice has been used in China for thousands of years to treat nephropathy, while the underlying mechanisms are still unclear. The triterpenoid and flavonoid compounds are the main components of licorice. The main bioactive components of licorice against nephropathy are glycyrrhizic acid, glycyrrhetinic acid, isoliquiritigenin, glabridin, isoliquiritin and licochalcone A. These components alleviate kidney injury through anti-inflammatory, anti-oxidative stress, anti-apoptosis, promoting autophagy, inhibiting EMT, and reducing ECM deposition by targeting the signaling pathways of NF-κB, TGF-β1, JNK, MAPK, p53, STAT3 and HMGB1. This review will provide a new insight to clarify the bioactive components of licorice against nephropathy and their corresponding mechanisms, which aims to provide useful information for the further application of licorice.

## 1 Introduction

Nephropathy refers to various disorders of the kidney, with clinical manifestations including proteinuria, hematuria, hypertension, and renal insufficiency (Javed et al., 2025). The average prevalence of chronic kidney diseases (CKD) is approximately 13.4% worldwide, and it shows an increasing trend year by year (Panzarino et al., 2022; Francis et al., 2024). The global morbidity rate of CKD is approximately 9.5%, and the mortality rate is approximately 2.66% (Bello et al., 2024; Xie et al., 2025). Without effective strategy, CKD will lead to end-stage renal disease and ultimately to renal failure, which will require the treatment with dialysis or kidney transplantation (August 2023; Vivante, 2024; Yan and Shao, 2024). However, it is difficult to find a suitable kidney donor. In addition, the cost is vast. Therefore, drug therapy may be a better choice. The drugs, used in clinic to treat kidney diseases such as the angiotensin-converting enzyme inhibitors and angiotensin II type 1 receptor blockers, show some side effects, which limit their clinical use (Na Takuathung et al., 2022; Zhu et al., 2022). Thus, drugs specific for kidney diseases should be developed. Many factors can lead to nephropathy, such as infection, hypertension, toxin, diabetes and obesity (Chen et al., 2021; Jung and Ihm, 2023; Hao and Lv, 2024). The damaged kidney often shows some disorders, including inflammation, oxidative stress, apoptosis, epithelial-mesenchymal transition (EMT), autophagy and the deposition of extracellular matrix (ECM) (Feng et al., 2020; Feng et al., 2021; Feng et al., 2022; Sanz et al., 2023; Srivastava et al., 2023; Rosales et al., 2024). Drugs alleviating these disorders will be therapeutic for nephropathy.

Nephropathy is primarily identified as urinary obstruction, severe urinary retention and nephrotoxicity in the theory of traditional Chinese medicine (TCM) (Lu et al., 2021; Wang et al., 2022). TCM shows a significant therapy effect on nephropathy by promoting blood circulation to remove blood stasis and clearing heat and dampness. Licorice is a key TCM in the treatment of nephropathy, while the underlying mechanisms are still unclear (Liu et al., 2018).

Licorice is derived from the dried roots and rhizomes of leguminous plants *Glycyrrhiza uralenssis* Fish., *Glycyrrhiza inflate* Bat. and *Glycyrrhiza glabra* L. (Wahab et al., 2021; Ding et al., 2022; Cui et al., 2023). It is mainly distributed in Inner Mongolia, Xinjiang and Gansu provinces in China. It has the functions of clearing heat and detoxifying, as well as expelling phlegm and relieving cough (Wu et al., 2010). The compounds derived from licorice not only exhibit renoprotective efficacy comparable to those of clinical drugs such as rosiglitazone, but also significantly cheaper, indicating a promising prospect for use as drugs (Tan et al., 2022).

In order to clarify the material basis and mechanisms of licorice in treating kidney diseases, this review focuses on the main components of licorice against kidney diseases and their corresponding mechanisms, which will provide useful information for its application in the treatment of nephropathy.

## 2 Chemical components of licorice

A great number of compounds have been isolated and characterized from licorice (Supplementary Table S1). These compounds are mainly composed of flavonoids, triterpenoids, coumarins, phenols, and alkanes constituents (Supplementary Table S1). The triterpenoids and flavonoids are the principal active constituents of licorice.

The triterpenoids are the most abundant constituents in licorice, predominantly existing as oleanane-type triterpenes (Supplementary Table S1). These compounds often show anti-inflammatory, antitumor, and antiviral activities. Glycyrrhizic acid is the most important bioactive compound of licorice.

Licorice is rich in flavonoid constituents with diverse structures, encompassing flavones, isoflavones, dihydroflavones, chalcones, and flavanones (Supplementary Table S1). Flavonoids often exhibit various pharmacological properties, such as anti-inflammatory, antioxidant, antibacterial, antiviral, and antitumor properties.

## 3 Mechanisms of licorice against nephropathy

There are many mediators contribute to the development of nephropathy, such as the signaling pathways of nuclear factor κ-light-chain-enhancer of activated B cells (NF-κB), transforming growth factor β1 (TGF-β1), signal transducer and activator of transcription 3 (STAT3), p53 and c-Jun N-terminal kinase (JNK). The compounds of licorice show a renoprotective effect on the damaged kidney by targeting these mediators. The following section compiles the mechanisms of action of selected active phytomolecules of licorice against nephropathy.

### 3.1 Inflammation

The inflammatory response is a defensive response of the body to tissue damage. Various cell types are involved in the inflammation response, including macrophages, T lymphocytes and B lymphocytes. Extensive studies reveal that the NF-κB and TGF-β1 signaling pathways play a key role in the progression of inflammation. When kidney is damaged, it can trigger inflammatory response. The early inflammation has a beneficial effect on tissue repair, while the chronic inflammation leads to kidney damage. Therefore, it is an effective strategy to suppress inflammation and alleviate kidney injury by inhibiting the NF-κB and TGF-β1 signaling pathways (Fearn et al., 2017).

#### 3.1.1 NF-κB signaling pathway

Both the extracts and the compounds of licorice suppress inflammation by inhibiting the NF-κB signaling pathway in the process of nephropathy.

The aqueous extract of licorice alleviated membranous glomerulonephritis-induced by cationic bovine serum protein via inhibiting the NF-κB signaling pathway in rats, suggesting that the highly polar compounds of licorice may exhibit the anti-inflammatory activity (Jin et al., 2016; Liu et al., 2019).

**FIGURE 1:**
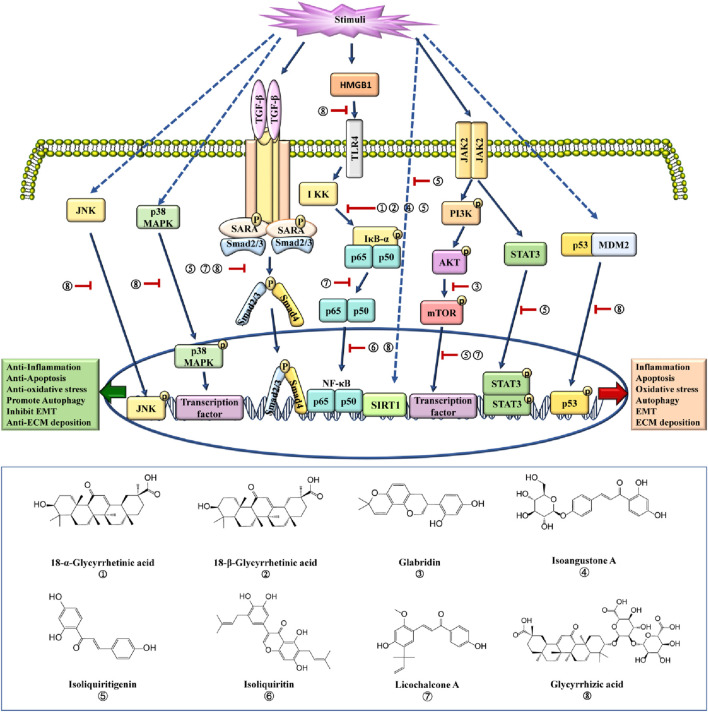
Mechanisms of licorice against nephropathy. AKT, protein kinase B; HMGB1, high mobility group box 1; IKK, IκB kinase; IκB-α, inhibitor of κB-α; JAK2, Janus kinase 2; JNK, c-Jun N-terminal kinase; MDM2, murine double minute 2; mTOR, mammalian target of rapamycin; NF-κB, nuclear factor κ-light-chain-enhancer of activated B cells; PI3K, phosphatidylinositol 3-kinase; SARA, Smad anchor for receptor activation; SIRT1, silent information regulator 1; Smad, mothers against decapentaplegic homolog; STAT3, signal transducer and activator of transcription 3; TGF-β, transforming growth factor β; TLR4, Toll-like receptor 4.

Multiple studies revealed that glycyrrhizic acid was the main anti-inflammatory component of licorice. It was reported that glycyrrhizic acid alleviated lipopolysaccharide (LPS)-induced inflammation in HBZY-1 cells and sepsis-induced inflammation in rats via inhibiting the NF-κB nuclear translocation (Figure 1; Table 1) (Zhao et al., 2016a; Zhao et al., 2016b). In addition, glycyrrhizic acid alleviated inflammation by down-regulating the NF-κB expression in acute kidney injury (AKI) mice (Table 1) (Wu et al., 2015). Moreover, glycyrrhizic acid protected against kidney injury via suppressing the NF-κB signaling pathway in insulin-resistant rats (Table 1) (Emara et al., 2021). Furthermore, glycyrrhizic acid attenuated inflammation and fibrosis-induced by high glucose via the AMPK signaling pathway in mice (Table 1) (Zhao et al., 2023). These results indicate that glycyrrhizic acid improves inflammation by inhibiting the NF-κB and MAPK signaling pathways.

**TABLE 1:** Licorice components against nephropathy.

Compound	Cellular mechanism	Model	Signaling pathway	Reference
Glycyrrhizic acid	Inflammation	LPS-treated HBZY-1 cells	NF-κB	Zhao et al. (2016a)
	Inflammation	Sepsis-treated rats	NF-κB	Zhao et al. (2016b)
	Inflammation	AKI mice	NF-κB	Wu et al. (2015)
	Inflammation	Insulin-resistant rats	NF-κB	Emara et al. (2021)
	Inflammation	High glucose-treated mice	AMPK	Zhao et al. (2023)
	Inflammation	Doxorubicin-treated rats	TGF-β1	Huang et al. (2009)
	ECM deposition	Adriamycin-treated rats	TGF-β1	Yu et al. (2010)
	Inflammation	Nicotine-induced podocyte injury	HMGB1	Datta et al. (2024a)
	Inflammation	Con A-treated mice	IL-25	Li et al. (2024)
	Oxidative stress	Cisplatin-treated HK-2 cells	p53	Ju et al. (2017)
	Oxidative stress	Cisplatin-treated mice	Unknown	Arjumand and Sultana (2011)
	Oxidative stress	Renal carcinoma cells	Unknown	Wu et al. (2013)
	Oxidative stress	AKI rats	HMGB1	Oh et al. (2021)
	Apoptosis	AKI mice	p38-MAPK	Ye et al. (2014)
	Autophagy	Tacrolimus-treated mice	Unknown	Cao et al. (2023)
	ECM deposition	Adriamycin-treated rats	Unknown	Wang et al. (2006)
	Other	Acute ischemia/reperfusion-treated rats	PI3K/AKT	Chen et al. (2023)
Glycyrrhetinic acid	ECM deposition	Diabetic nephropathy mice	Unknown	Li et al. (2018)
	EMT	Diabetic nephropathy mice	α-SMA/E-cadherin	Liu et al. (2020)
	Oxidative stress	Px-12-treated NRK-52E cells	JNK	Zhou et al. (2021)
	ECM deposition	Diabetic nephropathy rats	TGF-β1	Wang et al. (2025)
Isoliquiritigenin	Inflammation	Lipopolysaccharide-treated mice	IκB-α	Tang et al. (2018)
	Inflammation	Diabetic nephropathy mice	TLR4/NF-κB/NLRP3	Wang et al. (2024)
	Inflammation	Cisplatin-treated mice	Smad3/NF-κB	Wen et al. (2020)
	Inflammation	Diabetic nephropathy rats	SIRT1/NF-κB	Alzahrani et al. (2020)
	Inflammation	CKD mice	NF-κB	Liao et al. (2020)
	Inflammation	High-fat diet rats	TLR4/NF-κB	Yahya and Alshammari (2024)
	Inflammation	AKI mice	FPR2	Rui-Zhi et al. (2022)
	Inflammation	CKD rats	JAK2/STAT3	Atteia et al., 2023; Zhang et al. (2024)
	Oxidative stress	Adriamycin-treated rats	SIRT1	Huang et al. (2020)
	Autophagy	Caki cells	JAK2/STAT3	Kim et al. (2017)
	Autophagy	AKI mice	Unknown	Tang et al. (2021)
	ECM deposition	Diabetic nephropathy rats	TGF-β1	Li et al. (2010a)
	Autophagy	Renal carcinoma cells	PI3K/AKT/mTOR	Xin et al. (2019)
18β-Glycyrrhetinic acid	Inflammation	Ischemia/reperfusion injury-treated mice	IκB-α	Jiang et al. (2022)
	Oxidative stress	Diabetic nephropathy rats	Unknown	Alanazi et al. (2021)
	Oxidative stress	Bisphenol A-treated rats	JAK1/STAT1	Darendelioglu et al. (2025)
	Apoptosis	Cisplatin-treated HK-2 cells	BMP7	Ma et al. (2016)
	Other	Hemorrhagic shock rats	Unknown	Ge et al. (2024)
18α-Glycyrrhetinic acid	Inflammation	Excessive fructose-treated tubule epithelial cells	IκB-α	Cheng et al. (2020)
Isoliquiritin	Inflammation	Membranous glomerulonephritis rats	NF-κB	Liu et al. (2019)
Licochalcone A	Inflammation	AKI mice	IκB-α	Hu and Liu (2016)
	Other	Diabetic nephropathy mice	TGF-β1	Luo et al. (2021)
	Autophagy	Renal cancer 786-O and 769-P cells	PI3K/AKT/mTOR	Xin and Xu (2018)
	Other	Cisplatin-treated renal tubular epithelial cells	mTOR/HIF-1α	Wu et al. (2023)
Isoangustone A	Inflammation	High glucose-treated human mesangial cells	IκB-α	Li et al. (2011)
Glabridin	Inflammation	Contrast agent-treated rats	Unknown	Liu and Zhu (2018)
	Ferroptosis	Diabetic nephropathy rats	AKT	Tan et al. (2022)
	Other	Hemorrhagic shock-treated rats	Nrf2/HO-1	Shu et al. (2025)
Liquiritigenin	Inflammation	High glucose-treated HBZY-1 cells	Hippo/YAP/TAZ	Wen et al. (2024)
Glycyrrhizic acid glycosides	Other	Tripterygium glycosides-treated rats	RhoA/ROCK1	Zhou et al. (2024)

Isoliquiritigenin is the other anti-inflammation component of licorice. Isoliquiritigenin alleviated inflammation by inhibiting the phosphorylation of IκB-α in AKI induced by lipopolysaccharide in mice (Figure 1; Table 1) (Tang et al., 2018). Datta et al. reported that NOD-like receptor thermal protein domain associated protein 3 (NLRP3) could interact with thioredoxin-interacting protein to mediate podocyte injury-induced by nicotine (Datta et al., 2025). Isoliquiritigenin could improve inflammation and oxidative stress through suppressing the Toll-like receptor 4 (TLR4)/NF-κB/NLRP3 signaling pathway in mice with diabetic kidney disease (Table 1) (Wang et al., 2024). Moreover, isoliquiritigenin reduced inflammatory cell infiltration via inhibiting the mothers against decapentaplegic homolog 3 (Smad3)/NF-κB signaling pathway in AKI-induced by cisplatin in mice (Table 1) (Wen et al., 2020). Furthermore, isoliquiritigenin alleviated inflammation and collagen deposition through the silent information regulator 1 (SIRT1)/NF-κB signaling pathway in rats with diabetic nephropathy (Table 1) (Alzahrani et al., 2020). In addition, isoliquiritigenin alleviated inflammation and interstitial fibrosis by inhibiting the NF-κB signaling pathway in CKD mice (Table 1) (Liao et al., 2020). Isoliquiritigenin inhibited inflammation by the TLR4/NF-κB signaling pathway in high-fat diet rats (Table 1) (Yahya and Alshammari, 2024). From these cases, it can conclude that isoliquiritigenin alleviate renal inflammation by inhibiting the NF-κB signaling pathway.

Some other compounds of licorice also show an anti-inflammatory effect by regulating the NF-κB signaling pathway. Recent studies showed that both 18β-glycyrrhetinic acid and 18α-glycyrrhetinic acid alleviated inflammation by suppressing the phosphorylation of IκB-α in the process of nephropathy (Figure 1; Table 1) (Cheng et al., 2020; Jiang et al., 2022). In addition, isoliquiritin alleviated inflammation by inhibiting the NF-κB nuclear translocation in rats with membranous glomerulonephritis (Figure 1; Table 1) (Jin et al., 2016; Liu et al., 2019). Moreover, licochalcone A decreased the production of tumor necrosis factor-α (TNF-α), interleukin-6 (IL-6) and interleukin-1β (IL-1β) through inhibiting the IκB-α degradation in AKI mice (Figure 1; Table 1) (Hu and Liu, 2016). Moreover, isoangustone A alleviated inflammation and fibrosis by regulating the phosphorylation of IκB-α in human mesangial cells-treated with high glucose (Figure 1; Table 1) (Li et al., 2011).

In conclusion, licorice mainly improves renal inflammation by inhibiting the NF-κB signaling pathway and the key ingredients include glycyrrhizic acid, 18β-glycyrrhetinic acid, 18α-glycyrrhetinic acid, Isoliquiritigenin, isoliquiritin, licochalcone A, and isoangustone A.

#### 3.1.2 TGF-β1 signaling pathway

When the kidney is injured, the TGF-β1 signaling pathway is activated, thereby promoting inflammation, oxidative stress, autophagy, EMT and ECM deposition. The inhibition of TGF-β1 signaling pathway is the other mechanism of licorice exerting the anti-inflammatory effect. Recent study showed that glycyrrhizic acid alleviated doxorubicin-induced glomerular sclerosis by inhibiting the TGF-β1 signaling pathway in rats (Table 1) (Huang et al., 2009). In addition, glycyrrhizic acid improved the deposition of ECM and glomerular sclerosis by downregulating the TGF-β1 expression in rats (Figure 1; Table 1) (Yu et al., 2010). Moreover, licochalcone A significantly downregulated the TGF-β1 expression in mice with diabetic nephropathy (Figure 1; Table 1) (Luo et al., 2021). Furthermore, glycyrrhetinic acid alleviated renal fibrosis by targeting the TGF-β1 signaling pathway in rats with diabetic nephropathy (Figure 1; Table 1) (Wang et al., 2025).

In conclusion, the anti-inflammatory components of licorice via suppressing the TGF-β1 signaling pathway include glycyrrhizic acid, licochalcone A and glycyrrhetinic acid.

#### 3.1.3 Others

Licorice also shows the anti-inflammatory effects through other mechanism. High mobility group box 1 (HMGB1) shows a proinflammatory effect by binding with TLR4 in the development of many diseases (Datta et al., 2024b). Glycyrrhizic acid, a HMGB1 binder, could ameliorate the nicotine-induced podocyte injury via the downregulation of HMBG1 expression through TLR4 inactivation (Table 1) (Datta et al., 2024a). Glabridin significantly improved the histology structure of renal tubules and inflammatory cell infiltration in AKI-induced by contrast agent in rats (Table 1) (Liu and Zhu, 2018). In addition, liquiritigenin alleviated inflammation and fibrosis via regulating the Hippo/Yes-associated protein (YAP)/transcriptional co-activator with PDZ-binding motif (TAZ) signaling pathway in HBZY-1 cells-treated with high glucose (Table 1) (Wen et al., 2024). Moreover, glycyrrhizic acid alleviated renal inflammation via the Iinterleukin-25 (IL-25) signaling pathway in mice (Table 1) (Li et al., 2024). Furthermore, isoliquiritigenin alleviated renal inflammation by targeting formyl peptide receptor 2 (FPR2) in AKI mice (Table 1) (Rui-Zhi et al., 2022). In addition, isoliquiritigenin improved renal inflammation by inhibiting the Janus kinase 2 (JAK2)/STAT3 signaling pathways in CKD rats (Figure 1; Table 1) (Atteia et al., 2023; Zhang et al., 2024).

### 3.2 Oxidative stress

Oxidative stress is an imbalance between the production of intracellular or intercellular oxidants and the antioxidant defense system. Excessive ROS production cause oxidative stress. The p53 and STAT3 signaling pathways play an important role in oxidative stress (Charlton et al., 2020).

The aqueous extract of licorice alleviated the oxidative stress by inhibiting the STAT3 activation in AKI rats (Zhang et al., 2019). In addition, it was revealed that the aqueous extract of licorice and glycyrrhizic acid reduced reactive oxygen species (ROS) production by inhibiting the p53 signaling pathway in HK-2 cells-treated with cisplatin (Figure 1; Table 1) (Ju et al., 2017). Moreover, the aqueous extract of licorice alleviated AKI by eliminating oxygen-nitroso anions and inhibiting protein nitration in rats (Yokozawa et al., 2005). The aqueous extract of licorice alleviated AKI-induced by diosene through its antioxidant activity (Sakr et al., 2013). The aqueous extract of licorice alleviated gentamicin-induced AKI by scavenging oxygen free radicals, reducing lipid peroxidation and promoting antioxidant effects (Aksoy et al., 2012). In addition, the aqueous extract of licorice alleviated diabetic nephropathy through enhancing the kidney antioxidant capacity (Du et al., 2010). Moreover, the aqueous extract of licorice effectively increase the level of selenium glutathione peroxidase in 5/6 nephrectomy rats (Tang et al., 2018). These results indicate that the aqueous extract of licorice alleviates renal oxidative stress through multiple signaling pathways, involving the STAT3 and p53 signaling pathways.

The ethanol extract and the compounds of licorice also show a therapy effect on alleviating oxidative stress, but the mechanism remains to be further clarified. Kataya et al. reported that the ethanol extract of licorice reduced blood glucose level, improved renal function, and restored the activities of superoxide dismutase in rats with diabetic nephropathy (Kataya et al., 2011).

The main anti-oxidative stress components of licorice include ghlycyrrhizic acid, 18α-glycyrrhetinic acid, 18β-glycyrrhetinic acid and isoliquiritigenin. These compounds alleviate kidney injury through multiple mechanisms. Arjumand et al. reported that glycyrrhizic acid alleviated cisplatin-induced AKI through its antioxidant activity (Table 1) (Arjumand and Sultana, 2011). In addition, glycyrrhizic acid not only inhibited the proliferation of renal carcinoma cells, but also promoted the apoptosis of renal carcinoma cells through inhibiting oxidative stress (Table 1) (Wu et al., 2013). Moreover, glycyrrhizic acid showed a therapy effect on kidney injury by directly targeting HMGB1 in rats (Table 1) (Oh et al., 2021). Yao et al. demonstrated that glycyrrhetinic acid improved oxidative stress of renal tubular cells by regulating the JNK signaling pathway (Figure 1; Table 1) (Zhou et al., 2021). In addition, Cheng et al. found that 18α-glycyrrhetinic acid improved tubular epithelial cell injury by inhibiting oxidative stress, dyslipidemia and inflammation (Table 1) (Cheng et al., 2020). Ibtesam et al. revealed that 18β-glycyrrhetinic acid reduced ROS production and restored the antioxidant defenses in diabetic rats (Table 1) (Alanazi et al., 2021). Ekrem et al. showed that 18β-glycyrrhetinic acid alleviated kidney injury by inhibiting the Janus kinase 1 (JAK1)/signal transducer and activator of transcription 1 (STAT1) signaling pathway (Table 1) (Darendelioglu et al., 2025). Moreover, Xiaozhong et al. revealed that isoliquiritigenin alleviated oxidative stress by binding to SIRT1 in the process of kidney injury (Figure 1; Table 1) (Huang et al., 2020).

### 3.3 Apoptosis

Apoptosis is a programmed cell death, which is indispensable for development and tissue repair. A variety of biomolecules contribute to apoptosis, including bone morphogenetic protein-7 (BMP-7), p38, mitogen-activated protein kinase (MAPK) and caspase-3. The progression of nephropathy shows a close relation with the apoptosis of tubular epithelial cells. The aqueous extract of licorice improved apoptosis by inhibiting the caspase-3 signaling pathway in AKI cells (Chauhan et al., 2020). In addition, glycyrrhizic acid improved apoptosis and inflammation via suppressing the p38-MAPK signaling pathway in mice with AKI-induced by ischemia-reperfusion (Figure 1; Table 1) (Ye et al., 2014). Moreover, 18β-glycyrrhetinic acid inhibited the apoptosis via upregulating the BMP7 expression in HK-2 cells-treated with cisplatin (Table 1) (Ma et al., 2016). These results indicate that licorice improve apoptosis by regulating the BMP7, p38-MAPK and caspase-3 signaling pathways. The glycyrrhizic acid, 18β-glycyrrhetinic acid and licochalcone A are the main active components of licorice responsible for the anti-apoptosis activity.

### 3.4 EMT

Under pathological conditions, epithelial cells will lose their innate characteristics and acquire those of mesenchymal cells when the kidney is damaged, which is called EMT. EMT plays an important role in myofibroblasts origin. Myofibroblasts are the key effector cells causing ECM deposition in tissue gap and show a close relation with organ fibrosis and tumor. The Notch 2 signaling pathway plays a crucial role in the process of EMT, and inhibiting EMT is therapeutic for nephropathy.

Recent study showed that glycyrrhetinic acid downregulated the expressions of laminin-1 and α-smooth muscle actin (α-SMA) and upregulated the E-cadherin expression to alleviate fibrosis in mice with diabetic nephropathy (Table 1) (Liu et al., 2020). In addition, the methanol extract of de-glycyrrhizic acidated licorice improved EMT by inhibiting the Notch2 signaling pathway in NRK-52E cells-treated with high glucose, which indicated that some other components of licorice showed an inhibition effect on EMT except for glycyrrhetinic acid (Hsu et al., 2020).

### 3.5 Autophagy

Autophagy is a highly regulated process of organelle degradation and recycling. Some important signaling pathways are involved in the process of autophagy, including the signaling pathways of JAK2/STAT3, protein kinase B (AKT) and phosphatidylinositol 3-kinase (PI3K). Autophagy dysregulation can lead to cancer, and promoting autophagy in renal cancer cells can improve renal cancer.

Kim et al. demonstrated that isoliquiritigenin inhibited the proliferation of Caki cells by suppressing the JAK2/STAT3 signaling pathway (Figure 1; Table 1) (Kim et al., 2017). In addition, isoliquiritigenin upregulated autophagy-related protein 5 (ATG5) expression and downregulated the expressions of mammalian target of rapamycin (mTOR) and AKT in renal carcinoma cells, indicating that isoliquiritigenin exerted anti-cancer activity by inhibiting the PI3K/AKT/mTOR signaling pathway to induce autophagy (Figure 1; Table 1) (Xin et al., 2019). Furthermore, licochalcone A also induced autophagy by inhibiting the PI3K/AKT/mTOR signaling pathway in renal cancer 786-O and 769-P cells to alleviate renal carcinoma (Figure 1; Table 1) (Xin and Xu, 2018). In addition, Rui et al. found that glycyrrhizic acid attenuated kidney injury-induced by tacrolimus via regulating autophagy (Table 1) (Cao et al., 2023). Yun et al. showed that isoliquiritigenin improved AKI by regulating ferritinophagy-mediated ferroptosis in mice with AKI-induced by LPS (Table 1) (Tang et al., 2021).

### 3.6 ECM deposition

Under pathological conditions, the synthesis rate of ECM is greater than their degradation rate, which leads to ECM deposition and further affects organ function. ECM deposition is the main feature of renal fibrosis. It is crucial to reduce ECM deposition to delay CKD progression to renal failure. The activation of AKT and TGF-β1 signaling pathways contribute to ECM deposition. Therefore, inhibiting the AKT and TGF-β1 signaling pathways can alleviate renal fibrosis.

Li et al. showed that the aqueous and ethanol extracts of roasted licorice reduced ECM deposition by inhibiting the AKT and TGF-β1 signaling pathways in rats with diabetic nephropathy-induced by high glucose (Li et al., 2010b). Li et al. showed that isoliquiritigenin alleviated mesangial ECM deposition by inhibiting the TGF-β1 signaling pathway in rats with diabetic nephropathy-induced by high glucose (Table 1) (Li et al., 2010a). Glycyrrhizic acid improved glomerular sclerosis by reducing ECM deposition in rats (Table 1) (Wang et al., 2006). In addition, glycyrrhetinic acid reduced blood glucose and urinary creatinine levels and alleviate ECM deposition in mice with type 1 and type 2 diabetic nephropathy (Table 1) (Li et al., 2018). These cases indicate that the compounds of licorice such as isoliquiritigenin and glycyrrhizic acid may be the therapeutic drugs for renal fibrosis.

### 3.7 Others

Except for the above-mentioned mechanisms, licorice also improves kidney diseases through other pathways.

18β-Glycyrrhetinic acid reduced the levels of urea nitrogen and serum creatinine to alleviate kidney injury in rats with hemorrhagic shock (Table 1) (Ge et al., 2024). In addition, glabridin alleviated diabetic nephropathy by suppressing ferroptosis through the AKT signaling pathway in rat (Figure 1; Table 1) (Tan et al., 2022). Shu et al. reported that glabridin ameliorated hemorrhagic shock induced AKI through activating the Nrf2/HO-1 pathway in rat (Table 1) (Shu et al., 2025). Moreover, glycyrrhizic acid effectively reduced the levels of serum urea nitrogen, creatinine, malondialdehyde and lactate dehydrogenase in rats with acute ischemia/reperfusion renal injury. Glycyrrhizic acid protected against LPS-induced AKI through the PI3K/AKT signaling pathway in mice (Table 1) (Chen et al., 2023). It was reported that glycyrrhizic acid glycosides against tripterygium glycosides-induced nephrotoxicity by the Ras homolog family member A (RhoA)/Rho-associated coiled-coil containing protein kinase 1 (ROCK1) signaling pathway in rats (Table 1) (Zhou et al., 2024). Furthermore, licochalcone A protected renal tubular epithelial cell injury-induced by cisplatin via inhibiting the mTOR/hypoxia-inducible factor 1α (HIF-1α) signaling pathway (Table 1) (Wu et al., 2023).

## 4 Structure-activity relationships of the active components of licorice

**FIGURE 2:**
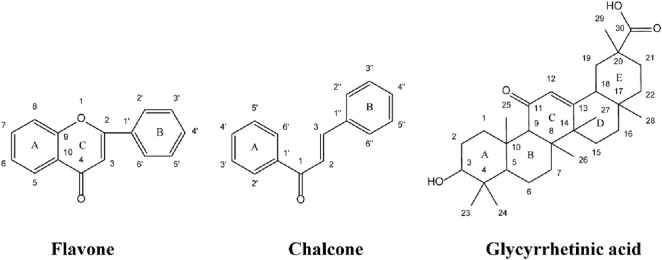
Chemical structures of flavone, chalcone and glycyrrhetinic acid.

The C3, C7 and C15 positions in glycyrrhizic acid and glycyrrhetinic acid are important for their anti-inflammatory activity (Figure 2). Ni et al. found that the different carbon chain lengths at C3 position of 18α-glycyrrhetinic acid could influence the inhibitory effect on NF-κB activation (Ni et al., 2022) (Figure 2). In addition, the hydroxylation at C7 or C15 of 18β-glycyrrhetinic acid could increase the anti-inflammatory activity. Furthermore, the introduction of different heterocycles conjugated with α, β-unsaturated ketones into ring A of 18β-glycyrrhetinic acid can increase apoptosis induction (Figure 2).

Glabridin shows low stability due to the pyranobenzene structure in ring A. Choi et al. found that 3″,4″-dihydro-glabridin exhibited better stability than glabridin (Figure 2) (Choi et al., 2021).

Lipase, 3β-hydroxysteroid dehydrogenases (3β-HSD), diacylglycerol acyltransferase and PPAR-γ show a close relationship with inflammation, oxidative stress, apoptosis, EMT, autophagy, and the deposition of ECM. Zeng et al. found that the isopentenyl and hydroxyl substituents at ring A of licorice chalcone were essential for the noncovalent inhibitory potency on pancreatic lipase (Figure 2) (Zeng et al., 2021). In addition, Choi et al. found that glabrol showed a potent inhibitory effect on diacylglycerol acyltransferase, which indicated that the prenyl group was the key structural feature responsible for the inhibitory effect (Choi et al., 2010). Moreover, Kuroda et al. found that the isoprenyl group at C6 and the C2’ hydroxyl group in the aromatic ring C of the isoflavan and isoflavene components of licorice are requirements for the binding activity on PPAR-γ (Figure 2) (Kuroda et al., 2003). Ye et al. found that the hydrogen bond acceptor in the licorice chalcone molecule was critical for the inhibitory effect on 3β-HSD1 and 3β-HSD2 (Ye et al., 2023). These results may be closely related to the renoprotective effect of licorice components.

## 5 Discussion

Licorice is a commonly used traditional Chinese medicine in the treatment of nephropathy in clinic. Modern pharmacology studies reveal that the extract and compound of licorice have a therapy effect on nephropathy. Although many compounds have been isolated from licorice, only a few of them have been studied for their nephroprotective effects. In this review, we summarized the existing studies on the nephroprotective effect of licorice. It was found that licorice showed its renoprotective effect through multiple mechanisms, including the properties of anti-inflammatory, anti-oxidative stress, anti-apoptosis, suppressing EMT, reducing ECM deposition and promoting autophagy. The core signaling pathways involved in the renoprotective effect of licorice include the signaling pathways of NF-κB, TGF-β1, STAT3, p53, Notch and PI3K/AKT/mTOR. In addition, both the aqueous and ethanol extracts of licorice have the renoprotective effect. The triterpene components of licorice, such as glycyrrhizic acid and glycyrrhetinic acid, as well as the flavonoids of licorice, including isoliquiritigenin, glabridin, isoliquiritin and licochalcone A, are the main active components of licorice in the treatment of nephropathy. Noteworthily, it was reported that glycyrrhetinic acid could lowered serum potassium concentration in hemodialysis patients (Farese et al., 2009). Therefore, the renoprotective effects of these compounds should be evaluated in clinic to promote them being as drugs.

Increasing evidences suggest that multitarget therapy may offer superior outcomes compared to single-target therapy in the treatment of kidney diseases (Voroneanu et al., 2025). Some components of licorice alleviated kidney diseases through multiple mechanisms. Hongyu Zhao et al. found that glycyrrhizic acid could simultaneously inhibited inflammation, apoptosis and oxidative stress in sepsis-induced kidney injury (Zhao et al., 2016a). In addition, Sanaa M Abd El-Twab et al. found that 18β-glycyrrhetinic acid could attenuate oxidative stress and inflammation to alleviate methotrexate-induced kidney injury (Abd El-Twab et al., 2016). Moreover, isoliquiritigenin mitigated diabetic kidney disease via oxidative stress and inflammation pathways (Wang et al., 2024). Furthermore, isoliquiritin ameliorated cisplatin-induced kidney injury by improving apoptosis, oxidative and inflammation (Pei et al., 2022). In addition, glabridin could reduce ROS content to alleviate inflammation in hemorrhagic shock-induced AKI (Shu et al., 2025). These results suggest that glycyrrhizic acid, 18β-glycyrrhetinic acid, isoliquiritigenin and glabridin are promising drugs in treating kidney diseases.

Licorice is considered a food-medicine homologous substance, with various benefits for human health (Hao et al., 2024). The mechanisms of licorice against kidney diseases are conducive to elucidating its benefits for kidney health, which will further facilitate the daily dietary application of licorice for patients with nephropathy.

Recently, it was demonstrated that the progression of nephropathy showed a deep association with miRNA, lnRNA and gut microbiota (Iatcu and Steen, 2021; Cerqueira et al., 2022; Yu et al., 2024). However, there is currently no study reporting whether licorice improve kidney injury through regulating the miRNA, lnRNA and gut microbiota, which limit the application of licorice. Thus, the further study of the mechanism of licorice against kidney injury should focus on miRNA, lnRNA and gut microbiota.

A larger number of compounds have been isolated and characterized from licorice. Among them, some may exhibit profound therapy effect on kidney injury. Molecular docking may be a powerful tool to screen the bioactive components of licorice against kidney injury. In addition, artificial intelligence has a significant advantage in screening active components, which saves time and costs in drug development. These powerful tools should be used in the pharmacology study of licorice. For screening the bioactive components from licorice compound prescriptions against kidney diseases, some suggestions are put forward. First, the bioactive components should be screened by activity-guided methods. Second, the network pharmacology prediction combined with experimental validation is effective to discover the bioactive components from licorice compound prescriptions. Third, reverse pharmacokinetics can be used to screen the components of licorice compound prescriptions that accumulate in the kidneys. These components may have renoprotective effects. Fourth, the different licorice components can be combined to target multiple targets simultaneously for nephropathy treatment, thereby identifying effective therapeutic strategies. Fifth, clinical studies on licorice for nephropathy treatment are insufficient, and it is suggested to conduct more clinical trials on licorice for nephropathy treatment.

Although licorice components show renoprotective potential, several obstacles must be overcome before they can be translated into the clinic. First, some licorice components show significant toxicity, such as glycyrrhizic acid, glycyrrhetic acid and isoliquiritigenin. The excessive intake of glycyrrhizic acid could cause mineralocorticoid excess syndrome by the targeting inhibition of 11β-hydroxysteroid dehydrogenase type 2 (11β-HSD2) in renal tissue (Makino, 2021; Blanpain, 2023; Caré et al., 2023). In addition, Lin et al. reported that isoliquiritigenin, licochalcone A, and licochalcone B also had a significant inhibitory effect on 11β-HSD2 (Lin et al., 2024). Moreover, Song et al. reported that isoliquiritigenin had developmental toxicity (Song et al., 2020). Second, glycyrrhetic acid is prone to degradation under acidic or photochemical conditions (Musharraf et al., 2013). Third, Zhu et al. found that drying significantly reduced the antioxidant activity of licorice, which might indicate that heat break the native chemical structures of licorice components (Zhu et al., 2025).
