# Supplementary material for: Licorice in nephropathy treatment: phytochemical compositions and pharmacological mechanisms
Source: Front Pharmacol. 2025 Nov 21;16:1672643. doi: 10.3389/fphar.2025.1672643 (PMC12678090; doi:10.3389/fphar.2025.1672643)
Supplement: Supplementary file 1 [file Table1.docx]

**Supplementary Table 1. Chemical components of licorice**

| **Number** | **Compound type** | **Compound** | **Molecule weight** | **Chemical structure** |
| --- | --- | --- | --- | --- |
| 1 | Flavone | Vicenin-2 | 594.57 |  |
| 2 | Flavone | Morusin | 420.49 |  |
| 3 | Flavone | Rutin | 610.57 |  |
| 4 | Flavone | Jaranol | 314.31 |  |
| 5 | Flavone | Nicotiflorin | 594.57 |  |
| 6 | Flavone | Neouralenol | 370.38 |  |
| 7 | Flavone | Isorhamnetin | 316.28 |  |
| 8 | Flavone | 7,4'-Dihydroxyflavone | 254.25 |  |
| 9 | Flavone | Narcissoside | 624.6 |  |
| 10 | Flavone | Kaempferol | 286.25 |  |
| 11 | Flavone | Glyasperin E | 444.51 |  |
| 12 | Flavone | Glepidotin A | 338.38 |  |
| 13 | Flavone | Licoflavone | 322.38 |  |
| 14 | Flavone | 7-Hydroxy-2-[4-hydroxy-3-(3-methylbut-2-enyl)phenyl]-6-(3-methylbut-2-enyl)chromone | 390.51 |  |
| 15 | Flavone | Licoflavonol | 354.38 |  |
| 16 | Flavone | Yinyanghuo D | 338.38 |  |
| 17 | Flavone | Licorice glycoside E | 693.71 |  |
| 18 | Flavone | 2-(3,4-Dihydroxyphenyl)-5,7-dihydroxy-6-(3-methylbut-2-enyl)chromone | 354.38 |  |
| 19 | Flavone | Gancaonin Q | 406.51 |  |
| 20 | Flavone | Vitexin | 432.41 |  |
| 21 | Flavone | Violanthin | 578.57 |  |
| 22 | Flavone | Schaftoside | 596.54 |  |
| 23 | Flavone | Nortangeretin | 302.25 |  |
| 24 | Flavone | Isoviolanthin | 578.57 |  |
| 25 | Flavone | Isoschaftoside | 564.54 |  |
| 26 | Flavone | 7-Acetoxy-2-methylisoflavone | 294.32 |  |
| 27 | Flavone | Artonin E | 436.49 |  |
| 28 | Flavone | Kanzonol E | 388.49 |  |
| 29 | Flavone | Astragalin | 448.41 |  |
| 30 | Dihydroflavone | (2R)-7-Hydroxy-2-[4-hydroxy-3-(3-methylbut-2-enyl)phenyl]chroman-4-one | 324.4 |  |
| 31 | Dihydroflavone | Xambioona | 388.49 |  |
| 32 | Dihydroflavone | Glyasperins K | 368.46 |  |
| 33 | Dihydroflavone | Glyasperins M | 368.41 |  |
| 34 | Dihydroflavone | 8-Prenylated eriodictyol | 356.4 |  |
| 35 | Dihydroflavone | 6-Prenylated eriodictyol | 356.4 |  |
| 36 | Dihydroflavone | 6″-O-Acetylliquiritin | 444.47 |  |
| 37 | Dihydroflavone | 3'(γ,γ-Dimethylallyl)-kievitone | 424.53 |  |
| 38 | Dihydroflavone | 3'-Hydroxy-4'-O-methylglabridin | 354.43 |  |
| 39 | Dihydroflavone | DFV | 256.27 |  |
| 40 | Dihydroflavone | Naringenin | 272.27 |  |
| 41 | Dihydroflavone | Pinocembrin | 256.27 |  |
| 42 | Dihydroflavone | 2',7-Dihydroxy-4'-methoxyisoflavan-7-O-β-d-glucopyranoside | 434.48 |  |
| 43 | Dihydroflavone | 3-Hydroxyglabrol | 408.53 |  |
| 44 | Dihydroflavone | (2S)-2-[4-Hydroxy-3-(3-methylbut-2-enyl)phenyl]-8,8-dimethyl-2,3-dihydropyrano[2,3-f]chromen-4-one | 390.51 |  |
| 45 | Dihydroflavone | Euchrenone | 406.56 |  |
| 46 | Dihydroflavone | Glyasperin B | 370.43 |  |
| 47 | Dihydroflavone | Glyasperin F | 354.38 |  |
| 48 | Dihydroflavone | Glyasperin C | 356.45 |  |
| 49 | Dihydroflavone | Glyasperins D | 370.48 |  |
| 50 | Dihydroflavone | Glyasperins Z | 340.45 |  |
| 51 | Dihydroflavone | Kanzonols X | 394.55 |  |
| 52 | Dihydroflavone | Naringin | 580.59 |  |
| 53 | Dihydroflavone | Glepidotin B | 340.4 |  |
| 54 | Dihydroflavone | Phaseolinisoflavan | 324.4 |  |
| 55 | Dihydroflavone | 3-(2-Hydroxy-4-methoxyphenyl)-2H-chromen-7-ol | 270.3 |  |
| 56 | Dihydroflavone | Glabrol | 392.53 |  |
| 57 | Dihydroflavone | (2R)-2-[3,4-Dihydroxy-5-(3-methylbut-2-enyl)phenyl]-5,7-dihydroxy-8-(3-methylbut-2-enyl)chroman-4-one | 424.53 |  |
| 58 | Dihydroflavone | Licoricidin | 424.58 |  |
| 59 | Dihydroflavone | Licoisoflavanone | 354.38 |  |
| 60 | Dihydroflavone | (E)-3-[3,4-Dihydroxy-5-(3-methylbut-2-enyl)phenyl]-1-(2,4-dihydroxyphenyl)prop-2-en-1-one | 340.4 |  |
| 61 | Dihydroflavone | Liquiritin | 418.43 |  |
| 62 | Dihydroflavone | 4H-1-Benzopyran-4-one, 2-(4-(beta-D-glucopyranosyloxy)phenyl)-2,3-dihydro-5,7-dihydroxy-, (2S)- | 434.43 |  |
| 63 | Dihydroflavone | Hispaglabridin A | 392.53 |  |
| 64 | Dihydroflavone | Sigmoidin-B | 356.4 |  |
| 65 | Dihydroflavone | Neoliquiritin | 418.43 |  |
| 66 | Dihydroflavone | (2R)-7-Hydroxy-2-(4-hydroxyphenyl)chroman-4-one | 256.27 |  |
| 67 | Dihydroflavone | (2S)-7-Hydroxy-2-(4-hydroxyphenyl)-8-(3-methylbut-2-enyl)chroman-4-one | 324.4 |  |
| 68 | Dihydroflavone | Liquiritin apioside | 550.56 |  |
| 69 | Dihydroflavone | Isograbrol | 392.53 |  |
| 70 | Isoflavone | Dehydroglyasperins C | 340.4 |  |
| 71 | Isoflavone | Odoratin | 314.31 |  |
| 72 | Isoflavone | Licoagroisoflavone | 336.36 |  |
| 73 | Isoflavone | Gancaonin H | 420.49 |  |
| 74 | Isoflavone | Gancaonin G | 352.41 |  |
| 75 | Isoflavone | 7-Hydroxy-2-methyl-3-phenyl-chromone | 252.28 |  |
| 76 | Isoflavone | Daidzein dimethyl ether | 282.31 |  |
| 77 | Isoflavone | Calycosin | 284.28 |  |
| 78 | Isoflavone | Lupiwighteone | 338.38 |  |
| 79 | Isoflavone | Formononetin | 268.28 |  |
| 80 | Isoflavone | 8-Prenylwighteone | 406.51 |  |
| 81 | Isoflavone | Castanin | 298.31 |  |
| 82 | Isoflavone | Hirsutrin | 464.41 |  |
| 83 | Isoflavone | 7-Methoxy-2-methyl isoflavone | 266.31 |  |
| 84 | Isoflavone | Kanzonols K | 436.54 |  |
| 85 | Isoflavone | Kanzonols L | 490.64 |  |
| 86 | Isoflavone | Kanzonols T | 440.53 |  |
| 87 | Isoflavone | Semilicoisoflavone B | 352.36 |  |
| 88 | Isoflavone | Licoricone | 382.44 |  |
| 89 | Isoflavone | Gancaonin A | 352.41 |  |
| 90 | Isoflavone | Gancaonin B | 368.41 |  |
| 91 | Isoflavone | Gancaonin C | 354.38 |  |
| 92 | Isoflavone | Prunetin | 284.28 |  |
| 93 | Isoflavone | Gancaonin D | 384.41 |  |
| 94 | Isoflavone | 3-(3,4-Dihydroxyphenyl)-5,7-dihydroxy-8-(3-methylbut-2-enyl)chromone | 354.38 |  |
| 95 | Isoflavone | 5,7-Dihydroxy-3-(4-methoxyphenyl)-8-(3-methylbut-2-enyl)chromone | 352.41 |  |
| 96 | Isoflavone | 5,7-Dihydroxy-3-(2-hydroxy-4-methoxy-phenyl)-6-(3-methylbut-2-enyl)chromone | 368.41 |  |
| 97 | Isoflavone | 3-[4,6-Dihydroxy-2-methoxy-3-(3-methylbut-2-enyl)phenyl]-7-hydroxy-chromone | 368.41 |  |
| 98 | Isoflavone | Licoisoflavone | 354.38 |  |
| 99 | Isoflavone | Licoisoflavone B | 352.36 |  |
| 100 | Isoflavone | Hispaglabridin B | 390.51 |  |
| 101 | Isoflavone | Glyzaglabrin | 298.26 |  |
| 102 | Isoflavone | Glabridin | 324.4 |  |
| 103 | Isoflavone | Glabranin | 324.4 |  |
| 104 | Isoflavone | Glabrene | 322.38 |  |
| 105 | Isoflavone | Glabrone | 336.36 |  |
| 106 | Isoflavone | Eurycarpin A | 338.38 |  |
| 107 | Isoflavone | Glycyroside | 562.57 |  |
| 108 | Isoflavone | Ononin | 430.44 |  |
| 109 | Isoflavone | Isoononin | 430.44 |  |
| 110 | Isoflavone | HMO | 268.28 |  |
| 111 | Chalcone | Licorice glycoside A | 726.74 |  |
| 112 | Chalcone | Corylifolinin | 324.4 |  |
| 113 | Chalcone | 1-(5-Hydroxy-2,2-dimethylchromen-6-yl)-3-(4-hydroxyphenyl)prop-2-en-1-one | 322.38 |  |
| 114 | Chalcone | 4,2',4',α-Tetrahydroxydihydrochalcone | 274.29 |  |
| 115 | Chalcone | 3,4,3',4'-Tetrahydroxy-2-methoxychalcone | 288.32 |  |
| 116 | Chalcone | Licochalcone A | 338.43 |  |
| 117 | Chalcone | (E)-1-(2,4-Dihydroxyphenyl)-3-(2,2-dimethylchromen-6-yl)prop-2-en-1-one | 322.38 |  |
| 118 | Chalcone | (E)-1-(2,4-Dihydroxyphenyl)-3-[4-hydroxy-3-(3-methylbut-2-enyl)phenyl]prop-2-en-1-one | 324.4 |  |
| 119 | Chalcone | Glyinflanin A | 408.53 |  |
| 120 | Chalcone | (2R)-1-[2,4-Dihydroxy-5-(3-methylbut-2-enyl)phenyl]-2-hydroxy-3-[4-hydroxy-3-(3-methylbut-2-enyl)phenyl]propan-1-one | 410.55 |  |
| 121 | Chalcone | (E)-1-[2,4-Dihydroxy-3-(3-methylbut-2-enyl)phenyl]-3-[4-hydroxy-3-(3-methylbut-2-enyl)phenyl]prop-2-en-1-one | 392.53 |  |
| 122 | Chalcone | Isoliquiritigenin | 256.27 |  |
| 123 | Chalcone | Glypallichalcone | 284.33 |  |
| 124 | Chalcone | Echinatin | 270.3 |  |
| 125 | Chalcone | Karenzu DK2 | 224.27 |  |
| 126 | Chalcone | Licochalcone B | 286.3 |  |
| 127 | Chalcone | Licochalcone C | 338.43 |  |
| 128 | Chalcone | LicochalconeD | 354.43 |  |
| 129 | Chalcone | (Z)-1-(2,4-Dihydroxyphenyl)-3-phenylprop-2-en-1-one | 240.27 |  |
| 130 | Chalcone | Licochalcone G | 354.43 |  |
| 131 | Chalcone | (E)-1-[2,4-Dihydroxy-3-(3-methylbut-2-enyl)phenyl]-3-(2,4-dihydroxyphenyl)prop-2-en-1-one | 340.4 |  |
| 132 | Chalcone | Neoisoliquiritin | 418.43 |  |
| 133 | Chalcone | Isoliquiritin | 418.43 |  |
| 134 | Chalcone | Licuraside | 550.56 |  |
| 135 | Flavonol | Gancaonin P | 370.38 |  |
| 136 | Flavonol | Uralenol | 370.38 |  |
| 137 | Flavonol | Uralenol-3-methylether | 384.41 |  |
| 138 | Flavonol | Uralene | 384.41 |  |
| 139 | Flavonol | Isolicoflavonol | 354.38 |  |
| 140 | Flavonol | Quercetin der. | 330.31 |  |
| 141 | Flavonol | Glyasperin A | 422.51 |  |
| 142 | Flavonol | Glycyrrhiza flavonol A | 370.38 |  |
| 143 | Flavonol | Kanzonol Z | 406.51 |  |
| 144 | Flavonol | Quercetin | 302.25 |  |
| 145 | Flavanes | 2-[(3R)-8,8-Dimethyl-3,4-dihydro-2H-pyrano[6,5-f]chromen-3-yl]-5-methoxyphenol | 338.43 |  |
| 146 | Flavanes | Licoriisoflavan A | 438.61 |  |
| 147 | Flavanes | Kanzonol H | 424.58 |  |
| 148 | Flavanes | Vestitol | 272.32 |  |
| 149 | Flavanes | (-)-Medicocarpin | 432.46 |  |
| 150 | Flavanes | 1-Methoxyphaseollidin | 354.43 |  |
| 151 | Flavanes | Medicarpin | 270.3 |  |
| 152 | Flavanes | Inermine | 284.28 |  |
| 153 | Flavanes | Licoagropin | 320.46 |  |
| 154 | Flavanes | 1-Methoxyficifolinol | 422.56 |  |
| 155 | Flavanes | Kanzonol F | 420.54 |  |
| 156 | Flavanes | Licoagrocarpin | 338.43 |  |
| 157 | Flavanes | Shinpterocarpin | 322.38 |  |
| 158 | Flavanes | 8-(6-Hydroxy-2-benzofuranyl)-2,2-dimethyl-5-chromenol | 308.35 |  |
| 159 | Oleanane triterpene | Mairin | 456.78 |  |
| 160 | Oleanane triterpene | Oleanolic acid | 456.78 |  |
| 161 | Oleanane triterpene | 18β-Glycyrrhetinic acid | 470.76 |  |
| 162 | Oleanane triterpene | Apioglycyrrhizin | 779.03 |  |
| 163 | Oleanane triterpene | Apioglycyrrhizin_qt | 470.76 |  |
| 164 | Oleanane triterpene | Liquoric acid | 484.74 |  |
| 165 | Oleanane triterpene | Glycyram | 823.04 |  |
| 166 | Oleanane triterpene | Licorice-saponin C2 | 807.04 |  |
| 167 | Oleanane triterpene | Licorice-saponin C2_qt | 454.76 |  |
| 168 | Oleanane triterpene | Licorice-saponin F3 | 983.18 |  |
| 169 | Oleanane triterpene | Licorice-saponin F3_qt | 454.76 |  |
| 170 | Oleanane triterpene | Glycyrrhizic acid | 823.04 |  |
| 171 | Oleanane triterpene | Uralsaponin B | 823.04 |  |
| 172 | Oleanane triterpene | Isoglabrolide | 468.74 |  |
| 173 | Oleanane triterpene | 22β-Acetylglabric acid | 528.8 |  |
| 174 | Oleanane triterpene | 24-Hydroxy-11-deoxyglycyrrhetic acid | 458.75 |  |
| 175 | Oleanane triterpene | 24-Hydroxyglycyrrhetic acid | 486.76 |  |
| 176 | Oleanane triterpene | 3β-Formylglabrolide | 496.75 |  |
| 177 | Oleanane triterpene | 11-Deoxyglycyrrhetic acid | 456.78 |  |
| 178 | Oleanane triterpene | Araboglycyrrhizin | 779.03 |  |
| 179 | Oleanane triterpene | Araboglycyrrhizin_qt | 470.76 |  |
| 180 | Oleanane triterpene | β-Glycyrrhetinic acid | 470.76 |  |
| 181 | Oleanane triterpene | 18α-Hydroxyglycyrrhetic acid | 486.76 |  |
| 182 | Oleanane triterpene | Licorice-saponin G2 | 839.04 |  |
| 183 | Oleanane triterpene | Licorice-saponin G2_qt | 486.76 |  |
| 184 | Oleanane triterpene | Licorice-saponin H2 | 823.04 |  |
| 185 | Oleanane triterpene | Licorice-saponin H2_qt | 470.76 |  |
| 186 | Oleanane triterpene | Licorice-saponin J2 | 825.06 |  |
| 187 | Oleanane triterpene | Licorice-saponin J2_qt | 472.78 |  |
| 188 | Oleanane triterpene | Licorice-saponin B2 | 809.06 |  |
| 189 | Oleanane triterpene | Licorice-saponin K2 | 823.04 |  |
| 190 | Oleanane triterpene | Licorice-saponin K2_qt | 470.76 |  |
| 191 | Oleanane triterpene | Glycyrrhetol | 456.78 |  |
| 192 | Oleanane triterpene | 3,22-Dihydroxy-11-oxo-delta(12)-oleanene-27-alpha-methoxycarbonyl-29-oic acid | 512.75 |  |
| 193 | Oleanane triterpene | Glabrolide | 468.74 |  |
| 194 | Oleanane triterpene | Ursolic acid | 456.78 |  |
| 195 | Simple coumarin | Scopoletol | 192.18 |  |
| 196 | Simple coumarin | Isotrifoliol | 298.26 |  |
| 197 | Furanocoumarin | Kanzonols W | 336.36 |  |
| 198 | Furanocoumarin | (2S)-6-(2,4-Dihydroxyphenyl)-2-(2-hydroxypropan-2-yl)-4-methoxy-2,3-dihydrofuro[3,2-g]chromen-7-one | 384.41 |  |
| 199 | Furanocoumarin | Licopyranocoumarin | 384.41 |  |
| 200 | Pyranocoumarin | Glycyrol | 366.39 |  |
| 201 | Pyranocoumarin | Glycycoumarin | 368.41 |  |
| 202 | Pyranocoumarin | Glycyrin | 382.44 |  |
| 203 | Isocoumarin | 1,3-Dihydroxy-9-methoxy-6-benzofurano[3,2-c]chromenone | 298.26 |  |
| 204 | Isocoumarin | 1,3-dihydroxy-8,9-dimethoxy-6-benzofurano[3,2-c]chromenone | 328.29 |  |
| 205 | Other coumarin | isoglycycoumarin | 368.41 |  |
| 206 | Other coumarin | Inflacoumarin A | 322.38 |  |
| 207 | Other coumarin | 7,2',4'-Trihydroxy-5-methoxy-3-arylcoumarin | 300.28 |  |
| 208 | Other coumarin | Phaseol | 336.36 |  |
| 209 | Other coumarin | 3-(2,4-Dihydroxyphenyl)-8-(1,1-dimethylprop-2-enyl)-7-hydroxy-5-methoxy-coumarin | 368.41 |  |
| 210 | Other coumarin | Isoglycyrol | 366.39 |  |
| 211 | Phenol | Protocatechuic acid | 154.13 |  |
| 212 | Phenol | Butylated hydroxytoluene | 220.39 |  |
| 213 | Phenol | Liconeolignan | 354.43 |  |
| 214 | Phenol | Gancaonin R | 382.54 |  |
| 215 | Phenol | Gancaonin S | 382.54 |  |
| 216 | Phenol | Gancaonin T | 398.54 |  |
| 217 | Phenol | Gancaonin U | 380.52 |  |
| 218 | Phenol | Gancaonin V | 312.39 |  |
| 219 | Phenol | Licocoumarone | 340.4 |  |
| 220 | Phenol | Gancaonin I | 354.43 |  |
| 221 | Phenol | Uralenneoside | 286.26 |  |
| 222 | Phenol | Docosyl caffeate | 488.83 |  |
| 223 | Aromatic | o-Xylene | 106.18 |  |
| 224 | Aromatic | m-Xylene | 106.18 |  |
| 225 | Aromatic | p-Xylene | 106.18 |  |
| 226 | Aromatic | EB | 106.18 |  |
| 227 | Aromatic | WLN: 4OVR | 178.25 |  |
| 228 | Aromatic | Anethole | 148.22 |  |
| 229 | Aromatic | Mipax | 194.2 |  |
| 230 | Aromatic | DIBP | 278.38 |  |
| 231 | Aromatic | DBP | 278.38 |  |
| 232 | Aromatic | 2-Ethyl-p-xylene | 134.24 |  |
| 233 | Hydrocarbon | Isoheptane | 100.23 |  |
| 234 | Hydrocarbon | Heptan | 100.23 |  |
| 235 | Hydrocarbon | 2,2-Dimethylpentane | 100.23 |  |
| 236 | Hydrocarbon | 2,3-Dimethylhexane | 114.26 |  |
| 237 | Hydrocarbon | (3S)-2,3-Dimethylpentane | 100.23 |  |
| 238 | Hydrocarbon | 2-Methyl-5-propyl -nonane | 184.41 |  |
| 239 | Hydrocarbon | OCT | 114.26 |  |
| 240 | Hydrocarbon | Methylheptane | 114.26 |  |
| 241 | Hydrocarbon | Octadiene | 110.22 |  |
| 242 | Hydrocarbon | (4S)-2,4-Dimethylhexane | 114.26 |  |
| 243 | Hydrocarbon | HEX | 86.2 |  |
| 244 | Hydrocarbon | (1S,2S)-1,2-Dimethylcyclopentane | 98.21 |  |
| 245 | Hydrocarbon | Sextone B | 98.21 |  |
| 246 | Hydrocarbon | Methylcyclopentane | 84.18 |  |
| 247 | Hydrocarbon | 21987_FLUKA | 136.26 |  |
| 248 | Hydrocarbon | β-Terpinene | 136.26 |  |
| 249 | Hydrocarbon | 2-Methyl-6-ethyl decane | 184.41 |  |
| 250 | Hydrocarbon | Isohexane | 86.2 |  |
| 251 | Hydrocarbon | (E)-Dodec-2-ene | 168.36 |  |
| 252 | Hydrocarbon | 3,3-Dimethylpentane | 100.23 |  |
| 253 | Hydrocarbon | 3-Methylheptane | 114.26 |  |
| 254 | Hydrocarbon | 3-Methylhexane | 100.23 |  |
| 255 | Hydrocarbon | 3-Methylpentane | 86.2 |  |
| 256 | Hydrocarbon | 3-Ethylpentane | 100.23 |  |
| 257 | Other | (L)-α-Terpineol | 154.28 |  |
| 258 | Other | Arachic acid | 312.6 |  |
| 259 | Other | α-Cubebol | 208.38 |  |
| 260 | Other | ICO | 161.17 |  |
| 261 | Other | Izoforon | 138.23 |  |
| 262 | Other | BuOH | 74.14 |  |
| 263 | Other | Sitosterol | 414.79 |  |
| 264 | Other | 2-Caren-10-al | 150.24 |  |
| 265 | Other | (E)-1-Butoxyhex-2-ene | 156.3 |  |
| 266 | Other | Glucuronic acid | 194.16 |  |
| 267 | Other | 5,6,7,8-Tetrahydro-2,4-dimethylquinoline | 161.27 |  |
| 268 | Other | Pentadecanol | 228.47 |  |
| 269 | Other | Cyclobutanol, 1-ethyl- | 100.18 |  |
| 270 | Other | 2-Tetradecanone | 212.42 |  |
| 271 | Other | 2,6,10-Trimethyl-dodecane | 144.14 |  |
| 272 | Other | 5,6,7,8-Tetrahydro-4-methylquinoline | 147.24 |  |
| 273 | Other | Icos-5-enoic acid | 310.58 |  |
| 274 | Other | 12-Methyltetradecanoate | 256.48 |  |
| 275 | Other | Gadelaidic acid | 310.58 |  |
| 276 | Other | Pentylfuran | 138.23 |  |
| 277 | Other | Menthol | 156.3 |  |
| 278 | Other | 2-Heptanone | 114.21 |  |
| 279 | Other | WLN: VH6 | 114.21 |  |
